# Atypical Cytomegalovirus-Associated Oral Ulcerations Mimicking Aphthous Lesions in a Child Receiving Blinatumomab: A Case Report

**DOI:** 10.7759/cureus.109363

**Published:** 2026-05-21

**Authors:** Soukayna Jebbar, Ghizlane El Amin, Noura Touyar, Amal Zouaki, Mohammed Hatimi, Mariame Lakhrissi, Maria Elkababri, Laila Hessissen, Hakima Kabbaj

**Affiliations:** 1 Central Virology Laboratory, Specialty Hospital - Ibn Sina University Hospital, Rabat, MAR; 2 Faculty of Medicine and Pharmacy of Rabat, Mohammed V University, Rabat, MAR; 3 Pediatric Hematology and Oncology Center, Children's Hospital - Ibn Sina University Hospital, Rabat, MAR

**Keywords:** b-cell acute lymphoblastic leukemia, cytomegalovirus, immunosuppression, polymerase chain reaction, ulcerative glossitis

## Abstract

Cytomegalovirus (CMV) is an opportunistic pathogen that can rarely cause ulcerative lesions of the oral mucosa in immunocompromised patients, although such manifestations often mimic other infectious, inflammatory, or traumatic etiologies. We report the case of a two-year-eight-month-old child with B-cell acute lymphoblastic leukemia undergoing blinatumomab therapy as a bridge to hematopoietic stem cell transplantation, who developed atypical, painless erosive glossitis. Clinical examination revealed polycyclic aphthoid lesions on the dorsum of the tongue in an afebrile, immunosuppressed patient. Virological polymerase chain reaction performed on lesion swabs and plasma detected CMV DNA, whereas tests for herpes simplex virus, varicella-zoster virus, Epstein-Barr virus, and enterovirus, as well as mycological microscopic examination, were negative.

These findings supported the diagnosis of CMV-associated ulcerative glossitis. The lesions resolved following discontinuation of blinatumomab and initiation of empirical antiviral and antifungal therapy. This case highlights the diagnostic challenges of CMV mucosal involvement in immunocompromised hosts, particularly in the absence of classic ulcerative features or confirmatory tissue biopsy. Detection of CMV in mucosal swabs requires careful interpretation, as asymptomatic shedding is common. Recognizing such atypical presentations is essential to guide appropriate diagnostic workup and therapeutic decision-making in high-risk pediatric oncology patients.

## Introduction

Human cytomegalovirus (CMV), also known as human herpesvirus 5, is a ubiquitous virus belonging to the *Herpesviridae* family. It is estimated that approximately 60-90% of adults worldwide have been exposed to CMV at some point in their lives, as evidenced by seropositivity for anti-CMV antibodies [1-4]. Viral transmission occurs through direct contact with infected body fluids, including saliva, breast milk, urine, semen, vaginal secretions, blood products, or transplanted organs and tissues [5]. Primary CMV infection typically occurs during childhood. Like all herpesviruses, CMV establishes lifelong latency with the potential for reactivation [5]. While CMV infection is often asymptomatic in immunocompetent individuals, it can cause a wide range of visceral manifestations in immunocompromised patients, potentially affecting the lungs, liver, gastrointestinal tract, central nervous system, and retina. Among these, mucocutaneous manifestations, particularly oral mucosal ulcerations, are rarely reported as signs of opportunistic CMV reactivation. Documented cases of CMV-related lesions in the oral cavity remain limited in the literature [2]. Here, we report a case of CMV infection presenting as atypical ulcerative glossitis in an immunocompromised child with acute lymphoblastic leukemia undergoing immunotherapy.

## Case presentation

A two-year-and-eight-month-old boy was followed at a pediatric hemato-oncology center for B-cell acute lymphoblastic leukemia. He was a candidate for a human leukocyte antigen-matched sibling hematopoietic stem cell transplantation. Initial treatment consisted of a polychemotherapy protocol, the Moroccan Acute Lymphoblastic Leukemia 2006 protocol (MARALL 06). Due to therapeutic failure, the patient was switched to biotherapy with blinatumomab, a bispecific T-cell engager (BiTE®) antibody, planned as three six-week cycles while awaiting allogeneic transplantation.

At the end of the third cycle of blinatumomab, the patient developed cytokine release syndrome immediately after initiation of the blinatumomab infusion, prompting discontinuation of treatment.

**Figure 1:**
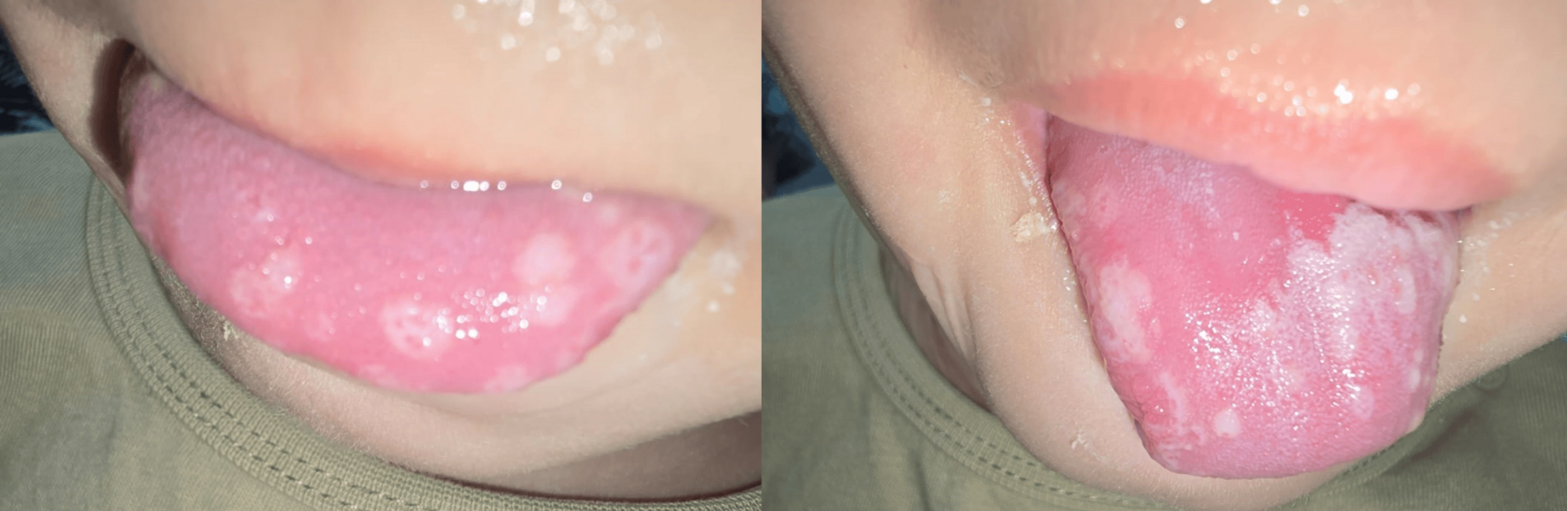
Atypical painless polycyclic erosions with a non-scrapable whitish coating on the dorsum of the tongue in an immunocompromised child undergoing blinatumomab therapy.

Two days later, he developed atypical painless aphthoid lesions on the dorsum of the tongue, characterized by multiple well-defined polycyclic erosions, approximately 1 mm in diameter, with an erythematous base and a non-scrapable white coating (Figure 1). No other oral mucosal involvement was noted, and no bite marks or other signs of trauma or thermal injury were observed on oral examination. These lesions did not affect hydration or nutritional status and occurred in an afebrile patient with preserved general condition.

Laboratory workup revealed leukopenia, with a white blood cell count of 3,400 cells/mm³, and severe lymphopenia at 200 cells/mm³ (Table 1). Samples were collected from the tongue lesions using synthetic nylon swabs for mycological and virological investigations. The patient was not receiving antiviral prophylaxis at the time of lesion onset. Empirical treatment with intravenous fluconazole, acyclovir, and intravenous immunoglobulin was initiated after sample collection.

**Table 1:** Summary of laboratory findings with reference ranges.

Parameter	Patient Value	Reference Range	Interpretation
White blood cell count	3,400 cells/mm³	5,000–15,000 cells/mm³ (child)	Leukopenia
Lymphocytes	200 cells/mm³	2,000–8,000 cells/mm³ (child)	Severe lymphopenia

Polymerase chain reaction (PCR) assays were performed on swab samples using the ELITe InGenius® system to detect herpes simplex virus type 1, herpes simplex virus type 2, varicella-zoster virus, CMV, Epstein-Barr virus, and enterovirus.

**Figure 2:**
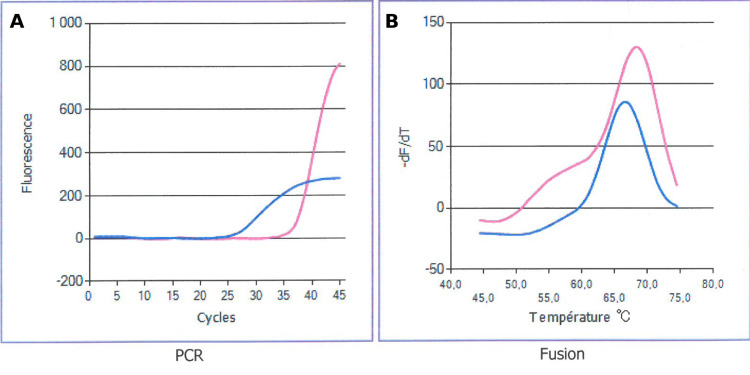
(A) Real-time PCR amplification curve and (B) melting curve demonstrating CMV DNA detection from an oral lesion swab. (A) The amplification curve shows a low-level positive signal for CMV DNA (red curve), with a cycle threshold (Ct) of 36.6, while the internal control (blue curve) confirms assay validity. (B) The melting curve demonstrates the specificity of the amplification product. CMV: Cytomegalovirus

PCR analysis of lesion swabs detected CMV DNA (Figure 2), while all other targets were negative. This finding was confirmed on a second sample collected a few days later, which again tested positive for CMV DNA.

A complementary plasma PCR was also positive, with a viral load of 838 IU/mL (2.92 log IU/mL).

CMV serology, performed on the Abbott Alinity system using a chemiluminescent microparticle immunoassay, showed negative IgM and positive IgG results, supporting probable viral reactivation in the appropriate clinical context.

Direct mycological examination did not reveal any yeast. Subsequent culture yielded low growth of Candida albicans. The low fungal load, inconsistent with candidal thrush, supports interpreting Candida as a commensal rather than an active pathogen.

The clinical course was marked by gradual resolution of the lingual lesions within a few days following discontinuation of blinatumomab and initiation of empirical antiviral and antifungal therapy, along with normalization of hematological parameters (white blood cell count: 5,800 cells/mm³; lymphocytes: 3,000 cells/mm³). This favorable course may suggest a contribution of immune reconstitution to clinical improvement.

## Discussion

Oral ulcerations are nonspecific lesions characterized by mucosal tissue loss and are frequently observed in congenital or acquired immunodeficiencies, autoimmune diseases, and iatrogenic immunosuppression, including drug-induced aplasia in transplant recipients and treatments such as chemotherapy or biotherapy agents (e.g., blinatumomab), as in our patient [1].

Historically, CMV-associated oral ulcerations were mainly described in human immunodeficiency virus infection in the acquired immunodeficiency syndrome stage, particularly with CD4 counts <100 cells/mm³ [6]. They are now more frequently reported in patients receiving immunosuppressive therapy, especially in the setting of organ transplantation [1].

The underlying mechanism involves CMV tropism for vascular endothelial cells, leading to cellular swelling and lysis, impaired microcirculation, tissue ischemia, and subsequent mucosal ulceration [2].

Studies have shown that CMV-related oral ulcers typically present as painful, yellowish, and extensive ulcerations with irregular or well-defined “die-cut” borders that extend deep into the tissue [6]. These lesions can be multiple and are often located on the mucosal surface of the lower lip, hard and soft palate, and tongue, causing significant pain, dysphagia, and dehydration. CMV ulcers tend to be deeper and larger than those caused by herpes simplex virus (HSV), which are usually smaller, rounded, and surrounded by a more prominent erythematous halo [5]. This clinical similarity with HSV-induced ulcers can complicate the diagnosis, highlighting the importance of careful evaluation and the added value of PCR testing in identifying the causative viral pathogen in immunocompromised patients [7]. In our patient, the lesions were small, superficial, polycyclic erosions with regular borders, clinically resembling herpetic lesions. However, they were painless and covered with a whitish coating, mimicking candidal thrush, although they were non-scrapable.

Alternative etiologies were also considered, including blinatumomab-related mucosal toxicity, chemotherapy-associated aphthous lesions, and immune-mediated mucositis. Isolated blinatumomab-related toxicity appeared less likely, as the oral lesions occurred only at the end of the third cycle, whereas previous cycles had not been associated with similar ulcerations. Nevertheless, a multifactorial mechanism cannot be excluded, given the patient’s profound immunosuppression and the concomitant detection of CMV DNA in both lesion swabs and plasma.

From a diagnostic perspective, according to the United States Department of Health and Human Services (HHS) guidelines [8], the diagnosis of CMV end-organ disease relies on two main criteria: the presence of compatible clinical manifestations and the detection of CMV in affected tissues [6]. Multiple diagnostic modalities are available, including serological tests (anti-CMV IgM and IgG), CMV DNA quantification in plasma by PCR, and histopathological or immunohistochemical (IHC) staining. Among these, plasma PCR is particularly useful for identifying active infection, while IHC performed on tissue biopsies allows for direct visualization of viral antigens [6]. Histopathological examination typically reveals cytopathogenic changes such as intranuclear and intracytoplasmic inclusions with the characteristic “owl’s eye” appearance [1]. Given that CMV-induced cellular changes are often localized in the deep connective tissue, biopsy is strongly recommended for accurate diagnosis [7]. These findings support the hypothesis that CMV may reach the oral mucosa via salivary gland excretion, subsequently infecting epithelial cells and fibroblasts, and leading to ulcerative lesions [6]. In our patient, the diagnosis was based on the detection of CMV DNA by PCR performed on direct swabs from the lingual erosions and on plasma samples. However, as salivary contamination and asymptomatic shedding are common in immunosuppressed patients, the specificity of swab PCR is limited [9]. Current literature recommends that a positive CMV swab in a symptomatic patient should prompt further clinical assessment but cannot alone confirm CMV pathogenicity. Additional confirmatory testing (e.g., blood PCR, biopsy, or IHC) is necessary before initiating antiviral treatment [9]. In our case, a biopsy was not performed, as it was deemed too invasive, particularly given the favorable clinical course. However, the concomitant detection of CMV DNA in both plasma and lesion swabs, together with compatible, albeit atypical, clinical findings and exclusion of other pathogens, strongly supports a probable CMV-related etiology.

The drugs of choice for CMV infections are ganciclovir and its oral prodrug valganciclovir. Although acyclovir and ganciclovir are both nucleoside analogues targeting viral DNA polymerase, CMV lacks thymidine kinase, which is required for acyclovir activation [10]. In contrast, ganciclovir is phosphorylated by the CMV-encoded UL97 kinase, allowing effective inhibition of viral DNA replication. As a result, acyclovir has minimal activity against CMV and is inadequate as monotherapy, particularly in immunocompromised patients [10]. In our case, clinical improvement was more likely attributable to the discontinuation of blinatumomab and immune reconstitution, as reflected by the normalization of hematological parameters, rather than to a direct antiviral effect of acyclovir. 

## Conclusions

This case highlights the diagnostic complexity of CMV-related oral ulcerations, as their etiology may be multifactorial, ranging from direct mucosal toxicity or drug-induced toxidermia (notably with agents such as blinatumomab) to opportunistic viral reactivation in the context of profound immunosuppression associated with acute lymphoblastic leukemia and its treatment. Establishing a definitive diagnosis requires a high index of clinical suspicion supported by targeted complementary investigations. Although CMV-induced oral ulcerations remain rare, clinicians, particularly oral health specialists, should be familiar with this entity in order to promptly identify and manage underlying immunodeficiency states. The gold standard for diagnosis relies on the detection of CMV in biopsy specimens, either by molecular techniques or histopathological and IHC analysis. The diagnostic hypothesis is further supported by the presence of CMV DNA in plasma in conjunction with a compatible clinical presentation.
